# Revisiting Estrogen: Efficacy and Safety for Postmenopausal Bone Health

**DOI:** 10.4061/2010/708931

**Published:** 2010-06-22

**Authors:** Sandra M. Sacco, Wendy E. Ward

**Affiliations:** Department of Nutritional Sciences, Faculty of Medicine, University of Toronto, Toronto, ON, Canada M5S 3E2

## Abstract

The rapid decline in endogenous estrogen production that occurs during menopause is associated with significant bone loss and increased risk for fragility fracture. While hormone therapy (HT) is an effective means to re-establish endogenous estrogen levels and reduce the risk of future fracture, its use can be accompanied by undesirable side effects such as stroke and breast cancer. In this paper, we revisit the issue of whether HT can be both safe and effective for the prevention of postmenopausal bone loss by examining standard and alternative doses and formulations of HT. The aim of this paper is to continue the dialogue regarding the benefits and controversies of HT with the goal of encouraging the dissemination of-up-to date evidence that may influence how HT is viewed and prescribed.

## 1. Introduction

Osteoporosis is a skeletal disorder characterized by low bone mass and low bone quality which leads to compromised bone strength and an increased risk of fracture [1]. The prevalence of osteoporosis in the United States and Canada is high, with 10 million Americans and 2 million Canadians affected with this disease. Several more millions, although not osteoporotic, suffer from low bone mass and will experience a fragility fracture in their lifetime [1, 2]. Along with its significant consequences to morbidity and mortality, osteoporosis has an annual cost of 20 billion dollars to the American and Canadian health care systems combined and, it is expected that this economic burden will rise due to rapidly aging populations [2, 3].

The decline in endogenous estrogen production that occurs during menopause has important implications for skeletal health because estrogen plays a major role in the development and maintenance of the human skeleton throughout the lifespan. In postmenopausal women, reduced levels of endogenous bioavailable estrogen are associated with lower bone mineral density (BMD) and higher risk for fragility fractures [4–7]. The repletion of endogenous estrogen through the use of hormone therapy (HT) effectively prevents postmenopausal bone loss and reduces the risk of fragility fractures [8–12]. 

While the benefit of HT, particularly conjugated equine estrogens (CEEs), on the prevention and treatment of postmenopausal osteoporosis and related fracture has been demonstrated, its effects on the health of other estrogen-sensitive tissues such as the uterus and breast must be carefully considered. In many circumstances, estrogen induces proliferation of the uterine and breast tissues [13–15]. In addition, postmenopausal women are at a higher risk for coronary heart disease (CHD) and breast cancer than their premenopausal counterparts, and the effects of HT may modify these risks. Moreover, estrogen has been shown to modulate vascular function; however, its effect on cardiovascular health is rather controversial. Evidence from observational trials [16, 17] suggests that HT is associated with a lower risk of CHD; however, subsequent data from two large randomized clinical trials (RCTs), the Heart and Estrogen/Progestin Replacement Study (HERS), and the Women's Health Initiative (WHI) trial did not confirm these benefits [9, 10, 18]. The HERS reported no benefit [18] from the use of HT, and the WHI study reported a significantly greater number of cardiovascular events in women taking HT compared to women taking placebo [9, 10]. Despite important limitations including high drop-out rates in the WHI, major consequences of the findings from WHI were a significant drop in HT prescription and use worldwide, with a concomitant increase in the prescription and use of alternative therapies (e.g., bisphosphonates) for the treatment of postmenopausal bone loss, for which their long-term consequences remain unknown [19, 20]. Many women were also left with little option for the relief of symptoms associated with the onset of menopause. 

Study findings including secondary analysis of the WHI have shown that HT is safe in younger postmenopausal women for the treatment of vasomotor symptoms and for the prevention of osteoporosis [21]. In fact, consensus statements from the North American and International Menopause Societies also support the notion that HT use may be safer in younger postmenopausal women (aged 50-59 years, or fewer than 10 years after the onset of menopause) [22–24]. In addition, RCTs are underway to confirm the safety of HT in younger postmenopausal women [25]. Nevertheless, long-term studies are needed to determine whether lower doses of HT with alternative modes of delivery are associated with fewer side effects compared to the standard HT formulations. 

In this paper, we revisit the usefulness of estrogen for the prevention and treatment of postmenopausal bone loss by reviewing the literature on its effectiveness at attenuating bone loss and risk of fracture and its side effects, in the context of alternative formulations and lower doses than traditionally prescribed. Through continuous dialogue and emerging evidence, the question of whether HT *should* be recommended to postmenopausal women is shifting to the question of *which type* of HT can be prescribed for the treatment of menopausal-related symptoms including bone loss, particularly in younger postmenopausal women.

## 2. Effects of Standard HT on Skeletal Health during Postmenopause

The most popular HT formulation prescribed to postmenopausal women is the CEE derived from urine of pregnant mares [26–28]. Medroxyprogesterone acetate (MPA) is conventionally added to CEE for women with intact uteri but, for hysterectomized women, CEE alone is used for the treatment of postmenopausal-related conditions including loss of bone mass. CEE, as indicated by its name, is itself made up of several conjugated forms of estrogen, mainly estrone sulfate and equilin sulfate. The most prescribed dose of CEE is 0.625 mg/d because this is the dose that alleviates postmenopausal-related symptoms in the majority of women that endure ovarian failure [29]. Other forms of estrogen in HT include esterified estrogen, ethinyl estradiol, 17*β*-estradiol, estradiol acetate, and estropipate. Norethindrone, levonorgestrel, and progesterone are the commonly used forms of progestogens in HT. 

Unquestionably, HT during postmenopause improves BMD and decreases fracture risk in the vertebrae as well as in nonvertebral sites including the hip (Table 1) [8–10, 12, 20, 31]. Observational studies have reported decreases of up to 71% in fracture risk with HT use [12, 20]. In a subgroup (*n* = 138,737) of postmenopausal women from the Million Women Study, current use of HT was associated with a 38% decrease in risk for total fractures, regardless of the type, dose, or delivery method of HT [20]. Similar observations were reported in other prospective cohorts including the Study of Osteoporotic Fractures trial, whereby current use of HT was associated with 34% and 38% decreases in risk for all nonspinal and hip fractures, respectively [31]. Moreover, current users that started HT within 5 years of menopause had a 50% decrease in risk for all nonspinal fractures and a 71% decrease in risk of hip fractures [31]. 

Similar to observational studies, RCTs have confirmed the protection of HT against fracture risk, albeit at more modest levels (~30%) (Table 1) [9, 10, 30]. The largest RCT reporting the effects of HT on fracture risk is the WHI. This trial was designed primarily to determine whether daily use of 0.625 mg CEE plus 2.5 mg MPA (for nonhysterectomized women) or 0.625 mg CEE alone (for hysterectomized women) reduces CHD in healthy postmenopausal women [9, 10]. Both the combination (CEE + MPA) and alone (CEE) arms were stopped early, after a mean of 5.2 and 6.8 years, respectively, because the number of observed side effects was deemed unacceptable. Nevertheless, CEE + MPA use was associated with a 34% decrease in risk for hip fractures and a 26% decrease in risk for total fractures [9]. Use of CEE alone was associated with a 39% decrease in risk for hip fractures and a 30% decrease in risk for total fractures (Table 1) [10]. 

While the positive effects of HT on bone health in postmenopausal women have been widely shown in both observational and clinical trials, the side effects popularized by its use in the WHI trial have resulted in dramatic decreases in its prescription and use worldwide [19, 26, 32]. Few women have been left with the option for HT use for simultaneously alleviating vasomotor symptoms and preserving bone tissue; however, the decision to discontinue HT may have been somewhat misguided.

## 3. Side Effects Associated with the Use of Standard HT

The potential for estrogen to modulate the health of many tissues in the body is based largely on the fact that estrogen receptors are located in both reproductive and nonreproductive tissues [33]. Thus, in addition to its ability to reduce vasomotor symptoms, vaginal atrophy and prevent postmenopausal bone loss, HT has the ability to modulate the health of many other tissues including the breast, liver, kidneys and cardiovascular tissues. Since postmenopausal women are at an increased risk for CHD and breast cancer, it is prudent to determine the safety profile of HT in these tissues. 

Described as an estrogen-deficiency disease, menopause was previously portrayed as a catalyst for the development of other diseases such as CHD, due to the dramatic decline in endogenous estrogen production [28, 34]. Evidence from observational trials indicated HT as a postmenopausal therapy with many health benefits, and that it was marketed to prolong youth and libido, resulting in its popularity to quickly expand worldwide [28]. To validate the notion that HT supports health during menopause and prevents the development of side-effects associated with other diseases, two large trials, the HERS and WHI, were carried out on a combined 30110 postmenopausal women in multicentre, randomized, double-blind, placebo-controlled designs [9, 10, 18]. The HERS trial was the first large RCT to evaluate whether daily use of 0.625 mg CEE plus 2.5 mg MPA alters the risk of CHD events in 2763 postmenopausal women with established CHD [18]. After an average follow-up of 4.1 years, HT did not confer protection against cardiovascular events (nonfatal myocardial infarction or CHD death), and its use was associated with higher venous thromboembolic events compared to placebo. Interestingly, HT use was associated with higher risk for CHD events during the first year of treatment, however, in the fourth and fifth years of the trial, its use was associated with lower risk for CHD events compared to placebo. Thus, the HERS trial demonstrated that the use of HT does not provide an overall benefit to cardiovascular health in women that have never used HT but it may provide benefit to cardiovascular health in current users that continue HT [18]. Subsequently, the WHI, which was the largest RCT to examine the effect of HT on the prevention of CHD, demonstrated that HT is associated with increased risk of CHD, stroke, venous thromboembolism, and breast cancer in the combination group and stroke in the estrogen alone group. The WHI findings resulted in early termination of the trial and subsequently widespread discontinuation of HT among postmenopausal women worldwide [9, 10, 26, 35]. 

The reasons for inconsistent findings among previous observational trials and the HERS and WHI trials may be due to differences in subject characteristics and inherent designs. For example, observational studies that have investigated the safety of HT have included younger postmenopausal subjects; however, the HERS and WHI trials consisted of mainly older postmenopausal women. In addition, observational studies can be subjected to unknown confounders that influence the outcome variable and are susceptible to a number of biases including sampling bias and admission rate bias, whereas RCTs can experience inadequate randomization, unblinding, and loss of sampling units— all of which can influence the outcome variable [36]. Nevertheless, since RCTs by design are superior compared to observational studies, the WHI study would inevitably have the greatest impact on HT recommendations for the postmenopausal population.

## 4. Limitations of the WHI

Important limitations of the WHI have become the focus of subsequent dialogue and have stimulated experts to question whether the WHI findings can be generalized to all postmenopausal women receiving various forms of HT. These limitations, which will be briefly discussed, include (i) a high loss of subjects, (ii) an older and healthy postmenopausal population included as subjects, and (iii) the investigation of one formulation, dose, and delivery method of HT. 

(i) The WHI suffered from a large loss of subjects. 42% of the subjects in the combination arm and 53.8% in the estrogen alone arm dropped out of the WHI resulting in reduced power of its design. The implication of a high drop-out rate may be an underestimation of observed effects, whether negative (ex: CHD and breast cancer) or positive (ex: fracture) [9]. However, being that losses in subjects are rarely a random occurrence [36], it is too simplistic to assume that the observed effects are merely an underestimation of what would have occurred if an appropriate number of subjects remained in the study throughout its total duration. 

(ii) The WHI enrolled into its trial mostly older and healthy postmenopausal women. While the WHI reported an unfavorable risk-to-benefit ratio, this study was conducted in a predominantly older and asymptomatic menopausal population, many of which who were 13–15 years past the onset of menopause. However, HT is targeted primarily to younger and more recently postmenopausal women for the treatment of menopausal symptoms including hot flushes, vaginal dryness, and decreased libido. Thus, there is a clear disconnect between the conventional target group of HT and the subjects from the WHI in terms of age and health status, and the question of whether similar findings would be observed in a younger postmenopausal population still remains. 

Observational studies point toward the notion that time since menopause may be an important factor on risk of CHD and other side effects of HT [24, 37]. Posthoc subgroup analysis by Rossouw et al. [21] of younger postmenopausal women (aged 50–59) in the WHI trials detected a tendency for HT to reduce the risk of CHD and total mortality. Further analysis of the WHI study by Manson et al. [38] determined that CEE alone resulted in lower coronary-artery calcification compared to placebo in the younger postmenopausal women aged 50–59 years. Nonetheless, these subsequent analyses were not adequately powered, especially in women aged 50–54 or closer to the onset of menopause (<5 years) [21]. Thus, difficulties remain in drawing clear conclusions from the secondary analyses of the WHI. While data from observational trials have shown that HT is protective against CHD, mortality, and other diseases in younger postmenopausal women, RCTs are underway to determine whether these previous findings can indeed be confirmed [25]. In the meantime, in March 2008, the International Menopause Society held a global summit to address the actual versus perceived risks of HT use in younger postmenopausal women [24]. Through the scrutiny of vast empirical evidence, this summit resulted in a consensus that there may be a “window of opportunity” whereby HT is safe in women that are closer to the onset of menopause (<10 years). Nevertheless, current opinion regarding the use of HT for the treatment of vasomotor symptoms and for the prevention of osteoporosis during the postmenopause is that the lowest effective dose should be used for the shortest duration possible.

(iii) Only two formulations of HT were investigated in the WHI, CEE + MPA (0.625 mg/d + 2.5 mg/d) for postmenopausal women with an intact uterus or CEE alone (0.625 mg/d) for postmenopausal women who had undergone hysterectomy. Both these formulations were provided as an oral tablet. Whether alternative formulations of HT, such as low-dose transdermal HT, result in fewer side effects than those observed with the HT used in the HERS or WHI trials is yet to be determined. However, compelling evidence points towards an advantage for lower doses of HT through the transdermal delivery method compared to oral standard dose HT. 

## 5. Effects of Lower Doses and Transdermal HT on Skeletal Health during Postmenopause

The most commonly prescribed HT for postmenopausal women is oral CEE (0.625 mg/d) alone or in combination with MPA (2.5 mg/d). In pursuit of the lowest effective dose for the attenuation of menopausal-related conditions including bone loss, several studies chose to examine doses that are three-quarters, half, and a quarter of the standard dose. In addition, alternative modes of delivery, such as the transdermal patch, are being studied because evidence indicates that they are associated with fewer side effects compared to the oral standard dose formulations. For the purpose of this paper, 0.3 mg and 0.45 mg CEE, along with 25 *μ*g transdermal 17*β*-estradiol are considered low-dose HT while 14 *μ*g transdermal 17*β*-estradiol is considered ultra-low-dose HT. 

Results from a number of trials have shown that lower doses of HT are effective in improving BMD in the hip and lumbar spine (Table 2) [39–47]. In a substudy (*n* = 822) of the Women's Health, Osteoporosis, Progestin, Estrogen (HOPE) trial, standard and lower doses of orally administered CEE alone or in combination with MPA resulted in significant improvements from baseline (1.33–3.46%) in spine and hip BMD, as well as biochemical markers of bone turnover in healthy postmenopausal women within 4 years since the onset of menopause [40]. These improvements contrasted from the placebo group, which experienced significant losses in spine BMD and total body bone mineral content. While CEE alone at the standard dose resulted in higher gains in spine BMD (2.43%) compared to CEE alone at the 0.3 mg/d dose (1.33%), the observed gains in BMD did not differ from the CEE alone at the 0.45 mg/d dose (2.09%). In addition, no differences in gains in hip BMD were observed between any of the treatment groups. Benefits in spine BMD of up to 3.5% have also been reported with the use of low-dose HT (0.3 mg/d CEE + 2.5 mg/d MPA) in older postmenopausal women with low bone mass [44].

The search for the lowest effective dose of HT has also been investigated using the transdermal delivery method [42, 47]. Using healthy postmenopausal women, both standard- and low-dose transdermal 17*β*-estradiol with 100 mg/d micronized progesterone resulted in similar increases in femur neck BMD (0.73% and 0.92%, respectively) after 18 months of treatment while the control group experienced a significant decrease in BMD (−2.23%) [42]. Another study observed improvements of 1.65% and 4.08% in spine BMD in healthy hysterectomized postmenopausal women that were treated for 2 years with unopposed standard- and low-dose transdermal 17*β*-estradiol, respectively [47]. Testing the effectiveness of unopposed ultra-low-dose transdermal 17*β*-estradiol, the Ultra-Low-dose Transdermal estRogen Assessment (ULTRA) observed a 2.6% increase in BMD at the lumbar spine in nonhysterectomized postmenopausal women after 2 years of treatment [39]. A non-hysterectomized sample was used since endometrial safety was also examined. Improvements in hip BMD and favorable changes in biochemical markers of bone turnover (osteocalcin and bone-specific alkaline phosphatase) were also observed from ultra-low-dose [39].

While no large head-to-head studies have been conducted between lower doses of HT and other pharmacologic agents used for the prevention or treatment of postmenopausal bone loss, ultra-low dose transdermal HT has also been shown to exert similar protection against bone loss in the spine as the selective estrogen receptor modulator, raloxifene [45]. In a 2-year RCT conducted in 500 osteopenic postmenopausal women, 77.3% of those treated with ultra-low-dose HT and 80.5% of those treated with raloxifene did not experience bone loss at the lumbar spine. Slight increases in total hip BMD were observed in both the ultra-low-dose HT and raloxifene groups, and raloxifene resulted in significantly greater improvements in hip BMD compared to ultra-low-dose HT. In addition, 63.8% of the ultra-low-dose HT and 81.3% of the raloxifene groups did not experience a loss of BMD at the hip. No difference in fracture incidence was observed between both groups but trials that are longer in duration are needed to adequately determine whether lower doses and alternative methods of delivery of HT modulate fracture risk [45]. 

## 6. Side Effects Associated with the Use of Lower Doses and Transdermal HT

Studies have shown that the dose and formulation of HT can be significant factors that determine the safety profile of HT. In the transdermal delivery system, 17*β*-estradiol is absorbed subcutaneously and is readily absorbed into tissues as it circulates systemically before reaching the liver. Because transdermal HT avoids first-pass liver metabolism, there is lower production of coagulation factors compared to oral formulations, which may result in a lower risk for cardiovascular events [48]. Thus, low-dose transdermal HT has the potential to provide significant benefit to skeletal health but with fewer side effects than the conventionally prescribed standard dose HT [49]. 

Evaluation of adverse events was included in the HOPE trial whereby a dose response in endometrial hyperplasia and vaginal bleeding was observed in women taking CEE alone, with the greatest number of reported adverse effects being in the 0.625 mg/d dose. A dose response in breast pain was observed in women taking CEE + MPA, with the greatest number of reported adverse effects being in the 0.625 mg/d dose [40]. The ULTRA trial, which examined the effects of unopposed ultra-low-dose transdermal 17*β*-estradiol on BMD and biochemical markers of bone turnover in non-hysterectomized postmenopausal women, did not observe higher incidences of endometrial hyperplasia compared to the placebo group [39]. In addition, further examination of the ULTRA trial by Grady et al. [50] demonstrated that nonopposed ultra-low-dose transdermal 17*β*-estradiol does not significantly change breast density in postmenopausal women. Nielsen et al. [51] observed that similar to raloxifene, no significant change in breast density in osteopenic postmenopausal women with 2-year treatment with ultra-low-dose transdermal 17*β*-estradiol. Whether no significant change in breast density from lower doses of ET ultimately results in reduced incidence of breast cancer compared to standard ET therapies is not yet known. Indeed, there is a necessity for long-term trials to assess whether lower doses of HT using alternative modes of delivery affect disease risk profiles.

## 7. Conclusion

The benefit of HT for the prevention and treatment of postmenopausal bone loss and for relief of vasomotor symptoms is well acknowledged. Thus, HT is an attractive option for the treatment of postmenopausal-related symptoms. However, due to its well-known ability to modulate physiology and subsequent disease risk in other tissues, the safety of HT must always be considered before it is prescribed. While standard-dose HT through the oral delivery method is effective against both postmenopausal bone loss and vasomotor symptoms, discrepancies in its safety have been noted between observational trials and RCTs. While review of the studies to date reveals discrepancies among studies and limitations of trials using standard doses of HT, emerging data suggests that HT can be safe for younger postmenopausal women. Moreover, lower doses of HT and alternative modes of delivery such as the transdermal patch may be a safe alternative to standard HT in protecting against postmenopausal bone loss while relieving vasomotor symptoms. The dialogue regarding HT should continue as findings from new studies using lower doses of HT and alternative models of delivery are published.

## Figures and Tables

**Table 1:** Selected studies on the effect of hormone therapy on bone metabolism in postmenopausal women

Study Name	Study Type	Sample Size, Mean Age in Years (Range), and Years since Menopause	Type of Therapy	Dose	Treatment Duration	Results for BMD, BMC, or Fracture Risk
WHI Estrogen plus Progesterone Trial [9]	RCT	16608, 63 (50–79), >6 months (>12 months for 50–54 years old)	CEE + MPA or placebo	CEE: 0.625 mg/d MPA: 2.5 mg/d (continuous)	Stopped after a mean of 5.2 years due to increases in breast cancer, stroke, CHD	HR, 0.66 [0.45–0.98] for incidence of hip fractures. HR, 0.76 [0.69–0.85] for incidence of total fractures.

WHI Estrogen alone Trial [10]	RCT	10739, 64 (50–79), previously hysterectomized	CEE or placebo	CEE: 0.635 mg/d	Stopped after a mean of 6.8 years due to increases in stroke	HR, 0.61 [0.41–0.91] for incidence of hip fractures. HR, 0.70 [0.63–0.79]for incidence of total fractures.

WISDOM Trial [30]	RCT	4385, 63 (50–69), >12 months or previously hysterectomized	CEE, CEE + MPA, or placebo	CEE: 0.635 mg/d MPA: 2.5 mg/d (continuous) or 5.0 mg/d (sequential)	Stopped after a mean of 11.9 months due to adverse findings inWHI [8]	HR, 0.69 [0.46-1.03] for incidence of osteoporotic fractures.

PEPI Trial [8]	RCT	875, 56 (45–64), 1 to <10 years (either naturally or surgically)	CEE, CEE + MPA, CEE + MP, or placebo	CEE: 0.625 mg/d MPA: 2.5 mg/d (continuous) or 10 mg/d (sequential) MP: 200 mg/d (sequential)	3 years	3.5–5.0% increase in LV BMD; 1.7% increase in hip BMD with these doses.

Danish Nurse Cohort Study [12]	Prospective	14015, 60 (≥50), Years since menopause not provided	All types (e.g., CEE, E_2_) and delivery methods (ex: oral, transdermal)	All Doses (ex: CEE, >0.625 mg/d or ≤0.625 mg/d; E_2_, >1 mg or ≤1 mg oral; E_2_, >50 *μ*g or ≤50 *μ*g transdermal); Various doses of MPA and NA as continuous or sequential regimes	Mean years of follow-up: not reported (Range: 0–6 years)	HR, 0.69 [0.50–0.94] for incidence of hip fractures with ever users.

Million Women Study [20]	Prospective	138737, Mean age in years not provided (50–69), Years since menopause not provided	All types (ex: CEE, E_2_) and delivery methods (ex: oral, transdermal)	All Doses (ex: CEE, >0.625 mg/d or ≤0.625 mg/d; E_2_, >1 mg or ≤1 mg oral; E_2_, >50 *μ*g or ≤50 *μ*g transdermal); Various doses of MPA and NA as continuous or sequential regimes	Mean years of follow-up: 2.8 (Range: 1.9–3.9 years)	RR, 0.62 [0.58–0.66] for incidence of fracture with current users. RR, 1.07 [0.99–1.15] for incidence of fracture with past users.

SOF Trial [31]	Prospective	9704, 70–72 (>65), 2 years before onset of menopause to >10 years	All types of oral preparations	All doses of oral preparations	Mean years of follow-up: 2.5 (Range: 0.02–6.5 years)	RR, 0.60 [0.36–1.02] for incidence of hip fractures with current users. RR, 0.66 [0.54–0.80] for incidence of all nonspinal fractures with current users. RR, 0.29 [0.09–0.92] for incidence of hip fractures with current users who started HT within 5 years of menopause. RR, 0.50 [0.36–0.70] for incidence of all nonspinal fractures with current users who started HT within 5 years of menopause.

RCT: Randomized Control Trial, CEE: Conjugated Equine Estrogen, MPA: Medroxyprogesterone Acetate, MP: Micronized Progesterone, NA: Norethisterone Acetate, E_2_: 17*β*-estradiol, BMD: Bone Mineral Density, LV: Lumbar Vertebra, HR: Hazard Ratio, and RR: Relative Risk.

**Table 2:** Selected studies on the effect of lower doses of hormone therapy on bone metabolism in postmenopausal women.

Study Name	Study Type	Sample Size, Mean Age in Years (Range), and Years since Menopause	Type of Therapy	Dose	Treatment Duration	Results for BMD, BMC, or Fracture Risk
ULTRA Trial [39]	RCT	417, 67 (60–80), ≥5 years	E_2_ patch or placebo patch*	E_2_: 0.014 mg/day	2 years	2.1% greater LV BMD versus placebo.

HOPE Trial [40]	RCT	822, 51.6 (40–65), 1 to ≤4 years	CEE, CEE + MPA, or placebo*	CEE: 0.625, 0.45, or 0.3 mg/d MPA: 2.5 or 1.5 mg/d	2 years	1.33–3.46% increase in LV BMD; Approximately 1.5–3% increase in hip BMD; 1.03–1.74% increase in total BMC, with these doses.

Gambacciani et al, 2008 [41]	Open Trial	Sample size not provided, 57 (range not provided), ≥1 year	E_2_ + NA (oral) or no treatment*	1 mg E_2_ + 0.5 mg NA for 28 d or 0.5 mg E_2_ + 0.25 mg NA per day	2 years	2–5% increase in LV BMD; 1.8–2.8% increase in femur neck BMD, with these doses.

Garcia-Pérez et al, 2006 [42]	Transversal Study	136, 53 for 0.05 mg/d and placebo groups to 56 for 0.025 mg/d group (range not provided), ≥1 year	E_2_ patch + micronized progesterone, or placebo*	E_2_: 0.05 or 0.025 mg/d progesterone: 100 mg/d	18 months	0.73–0.92% increase in femur neck BMD; −0.35–0.87% change in LV BMD, with these doses.

Gambacciani et al, 2001 [43]	Open Trial	38,54 (45–56), ≥1 year	CEE + MPA*	CEE: 0.3 mg/d MPA: 2.5 mg/d	2 years	2.7% increase in LV BMD.

CEE: Conjugated Equine Estrogen, MPA: Medroxyprogesterone Acetate, NA: Norethisterone Acetate, E_2_: 17*β*-estradiol, BMD: Bone Mineral Density, BMC: Bone Mineral Content, and LV: Lumbar Vertebrae. *All subjects received additional supplementation with calcium alone or with vitamin D.
